# Discovery of orally active chalcones as histone lysine specific demethylase 1 inhibitors for the treatment of leukaemia

**DOI:** 10.1080/14756366.2020.1852556

**Published:** 2020-12-13

**Authors:** Yang Li, Ying Sun, Yang Zhou, Xinyang Li, Huan Zhang, Guojun Zhang

**Affiliations:** a Department of Hematology, Shengjing Hospital of China Medical University, Shenyang, China;; b Department of Oncology, Shengjing Hospital of China Medical University, Shenyang, China

**Keywords:** Histone lysine specific demethylase 1, chalcone, H3K9me1/2, leukaemia, MOLT-4 xenograft

## Abstract

Histone lysine specific demethylase 1 (LSD1) has emerged as an attractive molecule target for the discovery of potently anticancer drugs to treat leukaemia. In this study, a series of novel chalcone derivatives were designed, synthesised and evaluated for their inhibitory activities against LSD1 *in vitro*. Among all these compounds, **D6** displayed the best LSD1 inhibitory activity with an IC_50_ value of 0.14 μM. In the cellular level, compound **D6** can induce the accumulation of H3K9me1/2 and inhibit cell proliferation by inactivating LSD1. It exhibited the potent antiproliferative activity with IC_50_ values of 1.10 μM, 3.64 μM, 3.85 μM, 1.87 μM, 0.87 μM and 2.73 μM against HAL-01, KE-37, P30-OHK, SUP-B15, MOLT-4 and LC4-1 cells, respectively. Importantly, compound **D6** significantly suppressed MOLT-4 xenograft tumour growth *in vivo*, indicating its great potential as an orally bioavailable candidate for leukaemia therapy.

## Graphical Abstract

**Figure:**
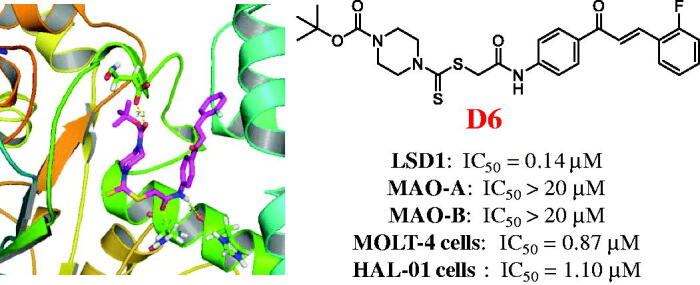

## Introduction

**Figure 1.**
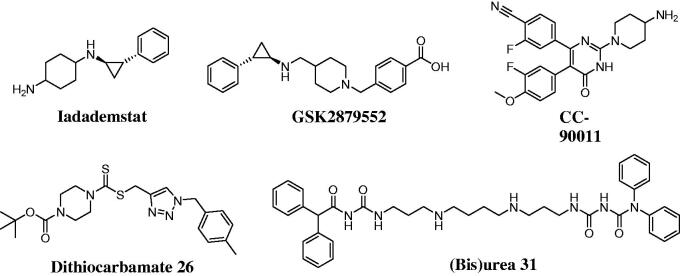
Chemical structures of LSD1 inhibitors.

Histone lysine specific demethylase 1 (LSD1) has been an epigenetic target for cancer therapy since its identification in 2004^1^. Aberrant over-expression of LSD1 is observed in various leukaemia cell lines and is closely associated with proliferation, migration and invasion^2–4^. These findings underscore the biological importance of LSD1 and therapeutic potential of LSD1 inhibitors for leukaemia therapy^5^. LSD1 inhibitors (**Iadademstat**, **GSK2879552** and **CC-90011**) have entered the clinical stages and are used to treat leukaemia (Figure 1)^6^. **Dithiocarbamate 26** and **(Bis)urea 31** as potent LSD1 inhibitors also effectively reduce the tumour growth against different human cancer cells^7^^,^^8^.

**Figure 2.**
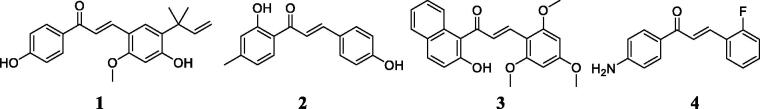
Chemical structures of anticancer chalcone derivatives.

Chalcones as 1,3-diaryl-2-propene-1-ones with the enone system between two aromatic rings possess a wide range of biological activities such as antibacterial, antioxidative, anticancer, antileishmanial, antiulcer, antiangiogenic, antiviral, immunosuppressive and anti-inflammatory activities^9–11^. More particularly, a number of synthetic and natural chalcones exhibited the potent anticancer activity against many cancer cell lines^12^^,^^13^. Chalcone **1** (Figure 2), a natural product isolated from the root of *Glycyrrhiza inflata*, could inhibit the drug transport function of ABCG2 and reverse ABCG2-mediated multidrug resistance in human multidrug-resistant cancer cell lines^14^. Chalcone **2** exhibited the reduction of tumour cell growth combined with inhibition of Notch1 intracellular domain^15^. Naphthalene-chalcone derivative **3** was found to induce significant cell cycle arrest at the G2/M phase and cell apoptosis against MCF-7 cell line^16^. Chalcone **4** displayed the potent antiproliferative activity against cancer cells by up-regulating the expression of P53 protein^17^.

**Figure 3.**
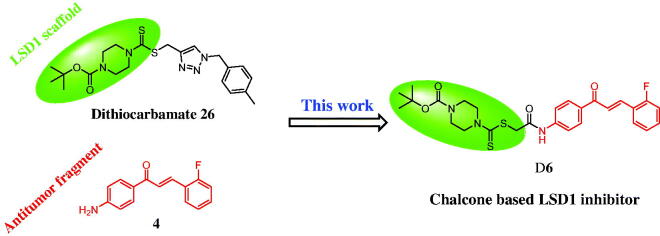
Rational design of chalcone based LSD1 inhibitors.

Molecular hybridisation is a new concept in drug design and development based on the combination of bioactive moieties of different compounds to produce a new hybrid with the improved affinity and efficacy^18^. These above interesting findings about LSD1 inhibitors and our continuous quest to identify more potently anticancer agents led to the molecular hybridisation of a LSD1 scaffold and an antitumor fragment to generate a new LSD1 inhibitor with the potentially anticancer activity. As shown in Figure 3, a molecular hybridisation strategy based on the structures of the reported LSD1 inhibitor **26** and antitumor agent **4** produced a scaffold that has three parts: (i) chalcone as an anticancer pharmacophore; (ii) a dithiocarbamate unit as the potential LSD1 moiety; (iii) an amide linker between chalcone and dithiocarbamate to form the hydrogen bond with LSD1. To the best of our knowledge, there have been few literature reports regarding anticancer chalcone derivatives as potent LSD1 inhibitors so far.

## Materials and methods

### General

Reagents and solvents were purchased (Innochem, Beijing, China). Melting points were determined on a micromelting apparatus (Tianjin XinZhou Science and Technology Co., Ltd, Tianjin, China). ^1^H NMR and ^13 ^C NMR spectra were recorded on a NMR spectrometer (DNP-NMR spectrometer, HuZhou Jingke Instrument Co., Ltd, FuZhou, China). High resolution mass spectra of all derivatives were recorded on a Waters Micromass Q-T of Micromass spectrometer by electrospray ionisation (Skyray Instrument, JiangShu, China).

### General procedure for the synthesis of compounds C1∼C4

Chalcone derivatives **B** were prepared by a condensation reaction from 1–(4-azidophenyl)ethan-1-one **A** and different benzaldehydes without purification. To a solution of triphenylphosphine (1 mmol), chalcone intermediates **B** (2 mmol), and tetrahydrofuran (12 ml) was added water (3 ml), the mixture was stirred for 4 h. Upon the completion, ethyl acetate and water were added. The organic layers were washed with water for several times to remove the tetrahydrofuran, and then evaporated to give the crude products. The crude product (1 mmol), chloroacetyl chloride (1.2 mmol), and triethylamine (0.5 mmol) were dissolved in acetone (10 ml) to stir for 8 h at room temperature. Upon completion, the system was purified with column chromatography (hexane: ethyl acetate = 9:1) to obtain analogues **C1∼C4**. Compound C4 was a reported chalcone intermediate from the previous reference [19].

#### (E)-2-Chloro-N-(4-cinnamoylphenyl)acetamide (C1)

Yellow solid, yield: 65%; m.p.: 145–147 °C. ^1^H NMR (400 MHz, CDCl_3_) δ 8.45 (s, 1H), 8.06 (d, *J* = 7.7 *Hz*, 2H), 7.82 (d, *J* = 15.8 *Hz*, 1H), 7.73 (d, *J* = 8.0 *Hz*, 2H), 7.65 (m, 2H), 7.53 (d, *J* = 15.8 *Hz*, 1H), 7.42 (m, 3H), 4.23 (s, 2H). ^13 ^C NMR (100 MHz, CDCl_3_) δ 188.96, 164.05, 144.87, 140.74, 134.87, 134.74, 130.62, 129.98, 129.00, 128.49, 121.68, 119.41, 42.90. HRMS (ESI) calcd. for C_17_H_15_ClNO_2_ [M + H]^+^: 300.0791, found: 300.0795.

#### (E)-2-Chloro-N-(4–(3-(3-fluorophenyl)acryloyl)phenyl)acetamide (C2)

Yellow solid, yield: 90%; m.p.: 156–158 °C. ^1^H NMR (400 MHz, CDCl_3_) δ 8.46 (s, 1H), 8.06 (d, *J* = 8.3 *Hz*, 2H), 7.83 − 7.67 (m, 3H), 7.54 (d, *J* = 15.8 *Hz*, 1H), 7.46 − 7.30 (m, 3H), 7.16 − 7.06 (m, 1H), 4.23 (s, 2H). ^13 ^C NMR (100 MHz, CDCl_3_) δ 188.54, 164.05, 143.30, 143.27, 140.90, 137.16, 134.44, 130.48, 130.00, 124.57, 122.81, 119.43, 117.30, 114.38, 42.89. HRMS (ESI) calcd. for C_17_H_13_ClFNO_2_ [M + H]^+^: 317.0619, found: 317.0623.

#### (E)-2-Chloro-N-(4–(3-(2-fluorophenyl)acryloyl)phenyl)acetamide (C3)

Yellow solid, yield: 86%; m.p.: 167–169 °C.^1^H NMR (400 MHz, DMSO-d_6_) δ 10.70 (s, 1H), 8.23 − 8.09 (m, 3H), 7.99 (d, *J* = 15.8 *Hz*, 1H), 7.89 − 7.77 (m, 3H), 7.57 − 7.49 (m, 1H), 7.33 (m, 2H), 4.34 (s, 2H). ^13 ^C NMR (100 MHz, DMSO-d_6_) δ 187.35, 165.21, 142.99, 134.64, 134.59, 132.51, 129.99, 129.06, 124.92, 124.89, 124.05, 124.00, 118.77, 115.93, 43.59. HRMS (ESI) calcd. for C_17_H_14_ClFNO_2_ [M + H]^+^: 318.0697, found: 318.0699.

### General procedure for the synthesis of compounds D1∼D7

To a solution of analogues **C1∼C4** (2 mmol) in acetone (20 ml) was added carbon disulphide (3 mmol), sodium phosphate dodecahydrate (1.5 mmol) and piperazine derivatives (2 mmol). The reaction mixture was stirred for 12 h. After the end of the reaction was established by TLC, the solvent was removed under vacuum, and excess saturated Na_2_CO_3_ solution was added. The resulted mixture was extracted with ethyl acetate, dried over MgSO_4_, filtered, and concentrated under vacuum. The product was purified by a silica gel column using ethyl acetate and petroleum ether as eluent to afford compounds **D1∼D7**. All the ^1^H NMR and ^13 ^C NMR spectra of compounds **D1∼D7** were listed in Supporting Information.

#### (E)-2-((4–(3-(4-Fluorophenyl)acryloyl)phenyl)amino)-2-oxoethyl-4-ethylpiperazine-1-carbodithioate (D1)

Yellow solid, yield: 72%; m.p.: 182–184 °C.^1^H NMR (400 MHz, CDCl_3_) δ 9.40 (s, 1H), 7.93 (d, *J* = 8.7 *Hz*, 2H), 7.69 (d, *J* = 15.7 *Hz*, 1H), 7.61 − 7.52 (m, 4H), 7.37 (d, *J* = 15.7 *Hz*, 1H), 7.04 (m, 2H), 4.33 (s, 2H), 4.20 (s, 2H), 3.92 (s, 2H), 2.50 (s, 4H), 2.40 (q, *J* = 7.2 *Hz*, 2H), 1.04 (t, *J* = 7.2 *Hz*, 3H). ^13^C NMR (100 MHz, CDCl_3_) δ 195.44, 188.67, 167.31, 143.09, 142.27, 133.67, 130.34, 130.25, 129.84, 121.57, 121.55, 119.17, 116.20, 115.98, 52.04, 51.78, 40.33, 11.94. HRMS (ESI) calcd. for C_24_H_27_FN_3_O_2_S_2_ [M + H]^+^: 472.1529, found: 472.1536.

#### (E)-2-((4-Cinnamoylphenyl)amino)-2-oxoethyl-4-methylpiperazine-1-carbodithioate (D2)

Yellow solid, yield: 92%; m.p.: 175–177 °C. ^1^H NMR (400 MHz, CDCl_3_) δ 9.47 (s, 1H), 8.01 (d, *J* = 8.7 *Hz*, 2H), 7.80 (d, *J* = 15.7 *Hz*, 1H), 7.69 − 7.63 (m, 4H), 7.53 (d, *J* = 15.7 *Hz*, 1H), 7.42 (m, 3H), 4.40 (s, 2H), 4.27 (s, 2H), 3.98 (s, 2H), 2.57 − 2.50 (m, 4H), 2.34 (s, 3H). ^13 ^C NMR (100 MHz, CDCl_3_) δ 195.65, 188.91, 167.28, 144.44, 142.21, 134.97, 133.76, 130.45, 129.87, 128.94, 128.42, 121.84, 119.15, 54.29, 45.54, 40.35. HRMS (ESI) calcd. for C_23_H_26_N_3_O_2_S_2_ [M + H]^+^: 440.1466, found: 440.1469.

#### (E)-2-((4-Cinnamoylphenyl)amino)-2-oxoethyl -4-ethylpiperazine-1-carbodithioate (D3)

Yellow solid, yield: 82%; m.p.: 181–183 °C. ^1^H NMR (400 MHz, CDCl_3_) δ 9.46 (s, 1H), 8.01 (d, *J* = 8.7 *Hz*, 2H), 7.80 (d, *J* = 15.7 *Hz*, 1H), 7.69 − 7.61 (m, 4H), 7.52 (d, *J* = 15.7 *Hz*, 1H), 7.42 (m, 3H), 4.40 (s, 2H), 4.27 (s, 2H), 3.99 (s, 2H), 2.57 (m, 4H), 2.47 (q, *J* = 7.2 *Hz*, 2H), 1.11 (t, *J* = 7.2 *Hz*, 3H). ^13 ^C NMR (100 MHz, CDCl_3_) δ 195.44, 188.92, 167.31, 144.44, 142.21, 134.98, 133.76, 130.44, 129.86, 128.94, 128.41, 121.85, 119.15, 52.04, 51.79, 40.32, 11.95. HRMS (ESI) calcd. for C_24_H_28_N_3_O_2_S_2_ [M + H]^+^: 454.1623, found: 454.1627.

#### (E)-2-((4–(3-(3-Fluorophenyl)acryloyl)phenyl)amino)-2-oxoethyl-4-methylpiperazine-1-carbodithioate (D4)

Yellow solid, yield: 85%; m.p.: 135–137 °C. ^1^H NMR (400 MHz, CDCl_3_) δ 9.40 (s, 1H), 7.95 (d, *J* = 15.7 *Hz*, 1H), 7.84 (d, *J* = 8.5 *Hz*, 2H), 7.57 (m, 4H), 7.32 (s, 1H), 7.10 (m, 2H), 4.32 (s, 2H), 4.19 (s, 2H), 3.91 (s, 2H), 2.47 (s, 4H), 2.27 (s, 3H). ^13 ^C NMR (100 MHz, CDCl_3_) δ 195.88, 194.63, 187.85, 166.26, 141.32, 136.10, 132.55, 131.84, 130.75, 128.93, 128.62, 123.45, 118.14, 118.00, 115.37, 53.25, 44.49, 39.32, 25.41. HRMS (ESI) calcd. for C_23_H_25_FN_3_O_2_S_2_ [M + H]^+^: 458.1372, found: 458.1378.

#### (E)-2-((4–(3-(3-Fluorophenyl)acryloyl)phenyl)amino)-2-oxoethyl-4-ethylpiperazine-1-carbodithioate (D5)

Yellow solid, yield: 90%; m.p.: 136–138 °C. ^1^H NMR (400 MHz, CDCl_3_) δ 9.40 (s, 1H), 7.95 (d, *J* = 15.7 *Hz*, 1H), 7.84 (d, *J* = 8.5 *Hz*, 2H), 7.57 (m, 5H), 7.32 (s, 1H), 7.10 (m, 1H), 4.32 (s, 2H), 4.19 (s, 2H), 3.91 (s, 2H), 2.51 (s, 4H), 2.45 (q, *J* = 7.2 *Hz*, 2H), 2.27 (t, *J* = 7.2 *Hz*, 3H). ^13 ^C NMR (100 MHz, CDCl_3_) δ 195.87, 194.43, 187.85, 166.29, 159.43, 141.33, 136.08, 132.55, 131.84, 130.75, 128.93, 123.45, 122.03, 118.00, 115.37, 51.00, 50.77, 39.30, 25.41, 10.89. HRMS (ESI) calcd. for C_24_H_27_FN_3_O_2_S_2_ [M + H]^+^: 472.1529, found: 472.1535.

#### Tert-butyl-(E)-4-(((2-((4–(3-(2-fluorophenyl)acryloyl)phenyl)amino)-2-oxoethyl)thio)carbonothioyl)piperazine-1-carboxylate (D6)

Yellow solid, yield: 75%; m.p.: 160–162 °C. ^1^H NMR (400 MHz, CDCl_3_) δ 9.31 (s, 1H), 7.94 (d, *J* = 8.7 *Hz*, 2H), 7.80 (d, *J* = 15.7 *Hz*, 1H), 7.61 − 7.54 (m, 4H), 7.32 (m, 1H), 7.09 (m, 2H), 4.30 (s, 2H), 4.21 (s, 2H), 3.90 (s, 2H), 3.53 (s, 4H), 1.41 (s, 9H). ^13 ^C NMR (100 MHz, CDCl_3_) δ 195.32, 187.87, 166.05, 161.96, 159.43, 153.29, 141.16, 136.18, 132.61, 130.69, 128.94, 123.49, 122.10, 118.15, 115.37, 115.15, 79.90, 51.14, 49.14, 39.29, 27.31. HRMS (ESI) calcd. for C_27_H_31_FN_3_O_4_S_2_ [M + H]^+^: 544.1740, found: 544.1747.

#### (E)-2-((4–(3-(2-Fluorophenyl)acryloyl)phenyl)amino)-2-oxoethyl-4-benzylpiperazine-1-carbodithioate (D7)

Yellow solid, yield: 81%; m.p.: 130–131 °C. ^1^H NMR (400 MHz, CDCl_3_) δ 9.40 (s, 1H), 7.94 (d, *J* = 8.7 *Hz*, 2H), 7.80 (d, *J* = 15.7 *Hz*, 1H), 7.61 − 7.54 (m, 4H), 7.31 (m, 1H), 7.24 (m, 5H), 7.10 (m, 2H), 4.33 (s, 2H), 4.19 (s, 2H), 3.91 (s, 2H), 3.50 (s, 2H), 2.54 (s, 4H). ^13 ^C NMR (100 MHz, CDCl_3_) δ 194.47, 187.85, 166.28, 161.95, 159.42, 141.27, 136.09, 132.53, 130.75, 128.92, 128.72, 128.18, 127.46, 126.62, 123.48, 123.33, 122.00, 118.13, 115.36, 61.24, 51.54, 49.47, 39.30. HRMS (ESI) calcd. for C_29_H_29_FN_3_O_2_S_2_ [M + H]^+^: 534.1685, found: 534.1689.

### Cell culture and cell viability assay

Cancer cell lines (Hela, 22RV1, Caco-2, BEL-7402, MOLT-4, OVCAR-3, HCT-8, HIC, IMR-32, HAL-01, KE-37, P30-OHK, SUP-B15 and LC4-1) were maintained in RPMI 1640 medium (Hyclone, Los Angeles, CA, USA) with 10% foetal bovine serum (Hyclone, Los Angeles, USA) and 1% penicillin-streptomycin (Hyclone, Los Angeles, USA) in a humidified atmosphere of 5% CO_2_ and 95% air at 37 °C. All cell lines were purchased from the China Centre for Type Culture Collection (CCTCC, China). Control containing sequence specific for LSD1 (GGCGAAGGTAGAGTACAGAGA) was described. The shRNA constructs were transfected into MOLT-4 cells using the Lonza Group nucleofector technology in accordance with the manufacturer’s instructions^20^. MOLT-4&shLSD1 cells and MOLT-4&shControl cells were also established and cultured according to the published references [21,22]. After the incubation for 24 h, cancer cell lines were cultured with the chalcone **D6** at different concentrations. Then, 20 μL of MTT (3–(4,5-dimethylthiazol-2-yl)-2,5-diphenyltetrazolium bromide) solution (5 mg/ml) was added and the cells were incubated for 4 h. The absorbance was measured using a microplate reader (DeTie Technology Co., Ltd, NanJing, China).

### LSD1, MAO-A/B and CDK1/2 enzyme assay

The inhibition of LSD1 activity was evaluated according to reported references [23,24]. pET-28b-LSD1 (full length) was transfected into BL21 (DE). Then, the protein was induced with 0.25 mmol/L IPTG following sonication and purified with Ni-NTA (Qiagen, Tubingen, Germany), Resource Q (GE, Pittsburgh, PA, USA) and Sephacryl S-200 HR (GE, Pittsburgh, PA, USA). The fluorescence intensity was read using EnVision Plate Reader (PerkinElmer, Waltham, MA, USA) to calculate the inhibition rate. The MAO-A and MAO-B were purchased from Active Motif (Cat#31502, Cat#31503, Carlsbad, CA, USA). Biochemical Kits were purchased from Promega (MAO-Glo Assay, Madison, WI, USA). The inhibitory activities of MAO-A and MAO-B were obtained according to the reported reference [25]. The inhibitory activities of CDK1 and CDK2 were obtained according to the reported reference [26].

### Dialysis assay

In the dialysis experiment, after incubation of the recombinant LSD1 and chalcone derivatives for 1 h at 37 °C, we dialysed the reaction system against 50 mmol/L HEPES buffer for 24 h at 4 °C and the reversibility was evaluated based on the activity of LSD1 in the dialysis tube.

### Dilution assay

LSD1 recombinant was incubated with the targeted compound, GSK-LSD1, or DMSO for 1 h. Then, the reaction system was diluted for 80 times. Finally, the above stated method was applied to detect the activity of LSD1 before and after dilution.

### The ultrafiltration experiment

In the ultrafiltration experiment, LSD1 recombinant was incubated with a concentration of 20-fold IC 50 inhibitor. The mixture was then added to a 10 kDa cut-off ultrafiltration tube (Millipore, Darmstadt, Germany) for centrifugation to remove the unbound compound. Finally, reversibility of the compound was evaluated by LSD1 assay for the upper chamber reaction system.

### Quantitative real-time PCR

Total RNA was isolated from MOLT-4&shControl and MOLT-4&shLSD1 cells with TRIzol reagent (Invitrogen, Carlsbad, CA, USA), the protocol was followed according to the manufacturer’s instructions, and RNA was quantified with Nanodrop (Tianjin XinZhou Science and Technology Co., Ltd, Tianjin, China). Quantitative real-time PCR assays were carried out on the Applied Biosystems QuantStudio™ Real-Time PCR detected system (Thermos Fisher, Waltham, MA, USA) using the Q-PCR kit with SYBR green dye (Vazyme Biotech, Nanjing, China). The primer sequences were as follows: LSD1 forward primer sequence (5′-3′), GTGGACGAGTTGCCACATTTC; LSD1 reverse primer sequence (5′-3′), TGACCACAGCCATAGGATTCC; gapdh forward primer sequence (5′-3′), GCACCGTCAAGGCTGAGAAC; gapdh reverse primer sequence (5′-3′), TGGTGAAGACGCCAGTGGA.

### Western blotting

Western blot was performed with the total lysates using RIPA buffer (Hyclone, Los Angeles, CA, USA). Same amounts of protein were subjected to SDS-PAGE, and then transferred to nitrocellulose membranes (PALL, Cortland, NY, USA). After blocking with 5% milk solution, the membranes were incubated at 4 °C with respective antibody overnight, followed by the incubation with a secondary antibody. Finally, the blot was visualised by enhanced chemiluminescence kit (Thermo Fisher, Waltham, MA, USA).

### Molecular docking studies

All molecular modelling studies were performed with the Autodock software (The Scripps Research Institute, San Diego, CA, USA). The crystal structure of LSD1 (PDB code: 5l3e) was downloaded from the RCSB protein database. The targeted compound was first generated using Pymol software. Following generation, the files were converted to the.pdbqt format using OpenBabel. It was then docked using AutoDockTools. The docked conformations and information were then docked and their resulting conformations were visualised using Pymol.

### Xenograft study

Animals were treated according to the protocols established by the ethics committee of Shengjing Hospital of China Medical University and the *in vivo* experiments were carried out in accordance with the approved guidelines and approved by the ethics committee of Shengjing Hospital of China Medical University. BALB/c nude mice were purchased from Hunan Slack Scene of Laboratory Animal Co., Ltd. (Hunan, China). Xenograft models using human leukaemia cells, MOLT-4, were established in BALB/c mice. Then, mice were separated into vehicle group and treatment groups (60 mg/kg and 100 mg/kg). The treatment groups received compound **D6** by intragastric administration for a period of 21 days.

## Results and discussion

### Chemistry

**Scheme 1.**
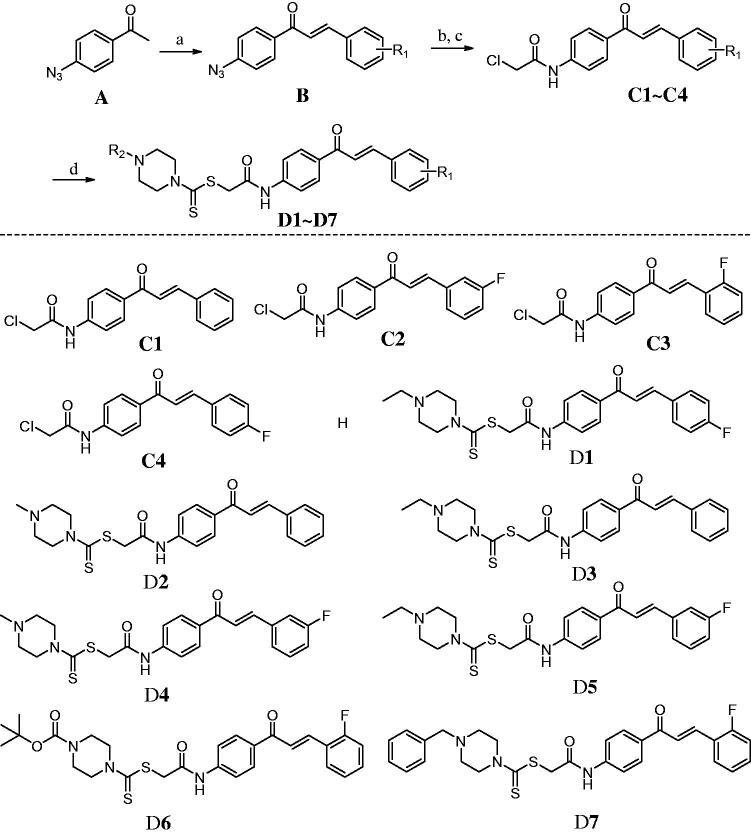
Synthesis of chalcones **D1**∼**D7**. Reagents and Conditions: (a) different benzaldehydes, NaOH, EtOH; (b) Ph_3_P, THF/H_2_O = 4: 1; (c) chloroacetyl chloride, Et_3_N, acetone; (d) CS2, piperazine derivatives, Na_3_PO_4_.12H_2_O, acetone.

A typical synthetic route for chalcone based LSD1 inhibitors is described in Scheme 1. Chalcone B was prepared by the condensation reaction of 1–(4-azidophenyl)ethan-1-one with different benzaldehydes. Intermediates **C1**∼**C4** were formed by the reduction reaction and acylation reaction. Next, the intermediates **C1**∼**C4** were reacted with carbon disulphide and piperazines under the presence of triethylamine to form chalcones **D1**∼**D7**.

### *In vitro* LSD1-inhibitory activity of chalcones C1∼C4 and D1∼D7

The LSD1 inhibitory activity of all synthesised compounds **C1**∼**C4** and **D1**∼**D7** was examined according to reported references [24,27]. Their results of inhibitory activities against LSD1 *in vitro* were summarised in Table 1. In this work, **Dithiocarbamate 26** and chalcone **4** were used as reference compounds. The replacement of the chlorine atom by the dithiocarbamate fragment resulted in a powerful improvement of LSD1 inhibitory activity for chalcone-dithiocarbamate derivatives **D1**∼**D7** compared with the corresponding chalcone analogues (**C1**∼**C4**). Especially, compound **D6** showed the potently inhibitory effect with an IC_50_ value of 0.14 μM (> 100-fold more potent than **C3**). This result suggests that dithiocarbamate moiety may play a synergistic role in determining activity.

**Table 1.** *In vitro* inhibitory activities of compounds **C1**∼**C4** and **D1**∼**D7** to LSD1 and its homologies MAO-A and MAO-B 
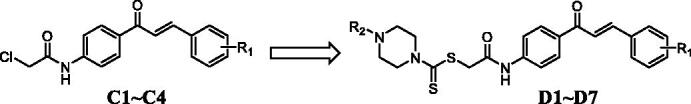

Compound	R1	R2	IC_50_ (μM)		
			LSD1	MAO-A	MAO-AB
**C1**	**H**	**-**	>20	>20	>20
**C2**	**3-F**	**-**	19.70 ± 0.67	>20	>20
**C3**	**2-F**	**-**	15.35 ± 0.28	>20	>20
**C4**	**4-F**	**-**	>20	>20	>20
**D1**	**4-F**	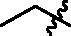	11.26 ± 0.13	>20	>20
**D2**	**H**	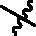	9.35 ± 0.14	>20	>20
**D3**	**H**	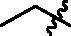	13.90 ± 0.18	>20	>20
**D4**	**3-F**	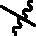	6.03 ± 0.17	>20	>20
**D5**	**3-F**	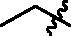	2.29 ± 0.35	>20	>20
**D6**	**2-F**	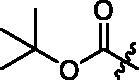	0.14 ± 0.01	>20	>20
**D7**	**2-F**	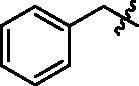	3.27 ± 0.13	>20	>20
**Dithiocarbamate 26** ^a^	**-**	**-**	2.68 ± 0.12	>20	>20
**Chalcone 4** ^a^	**-**	**-**	>20	>20	>20

^a^**Dithiocarbamate 26** and **Chalcone 4** were used as control.

**Scheme 2.**
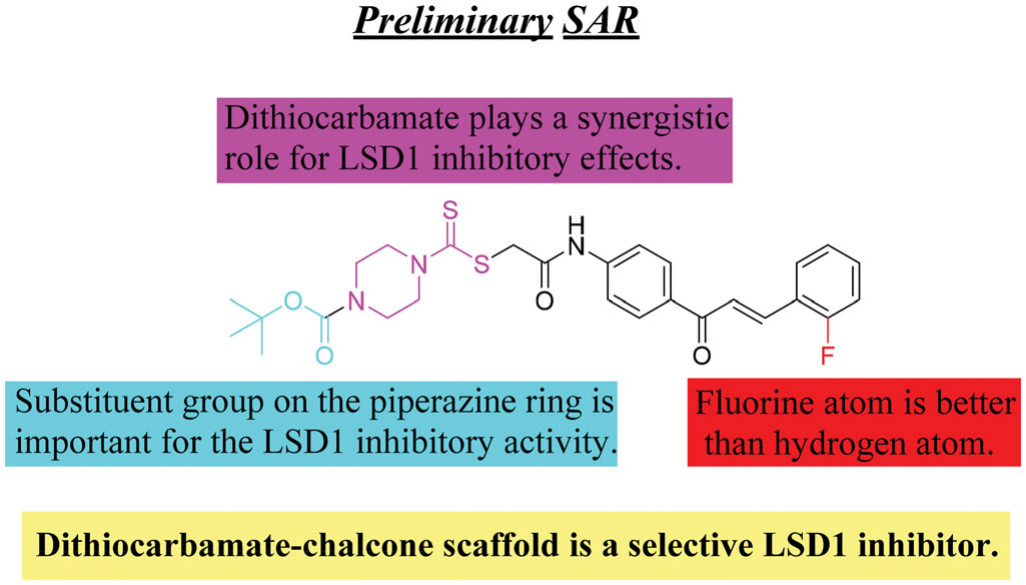
Summary of preliminary SAR.

As LSD1 belongs to the monoamine oxidase (MAO) family, the inhibitory effects of compounds **C1**∼**C4** and **D1**∼**D7** to its homologies MAO-A and MAO-B were also examined using commercially available kits^28^^,^^29^. From the results of Table 1, all synthesised compounds **C1**∼**C4** and **D1**∼**D7** had no significant effects on MAO-A and MAO-B. These findings indicated the high selectivity of chalcone-dithiocarbamate inhibitors **D1**∼**D7** on LSD1 *in vitro*. In addition, we found that the substitution on the phenyl ring was important for the activity showing an over 6-fold activity loss, when the fluorine atom was replaced with the hydrogen atom (compounds **D3** vs. **D5**). Replacement of the ethyl group of compound **D5** with a methyl group (**D4**) led to a loss of the activity. However, changing the benzyl group (compound **D7**) to a tert-butoxycarbonyl group (compound **D6**) led to a significant improvement of the activity against LSD1. All these results indicated that the substituent group at piperazine ring may play an important role for their inhibitory activity. The detailed illustration for preliminary structure activity relationship (SAR) of target derivatives was showed in Scheme 2.

### Antiproliferative effects of chalcone D6 against different cancer cell lines

**Figure 4.**
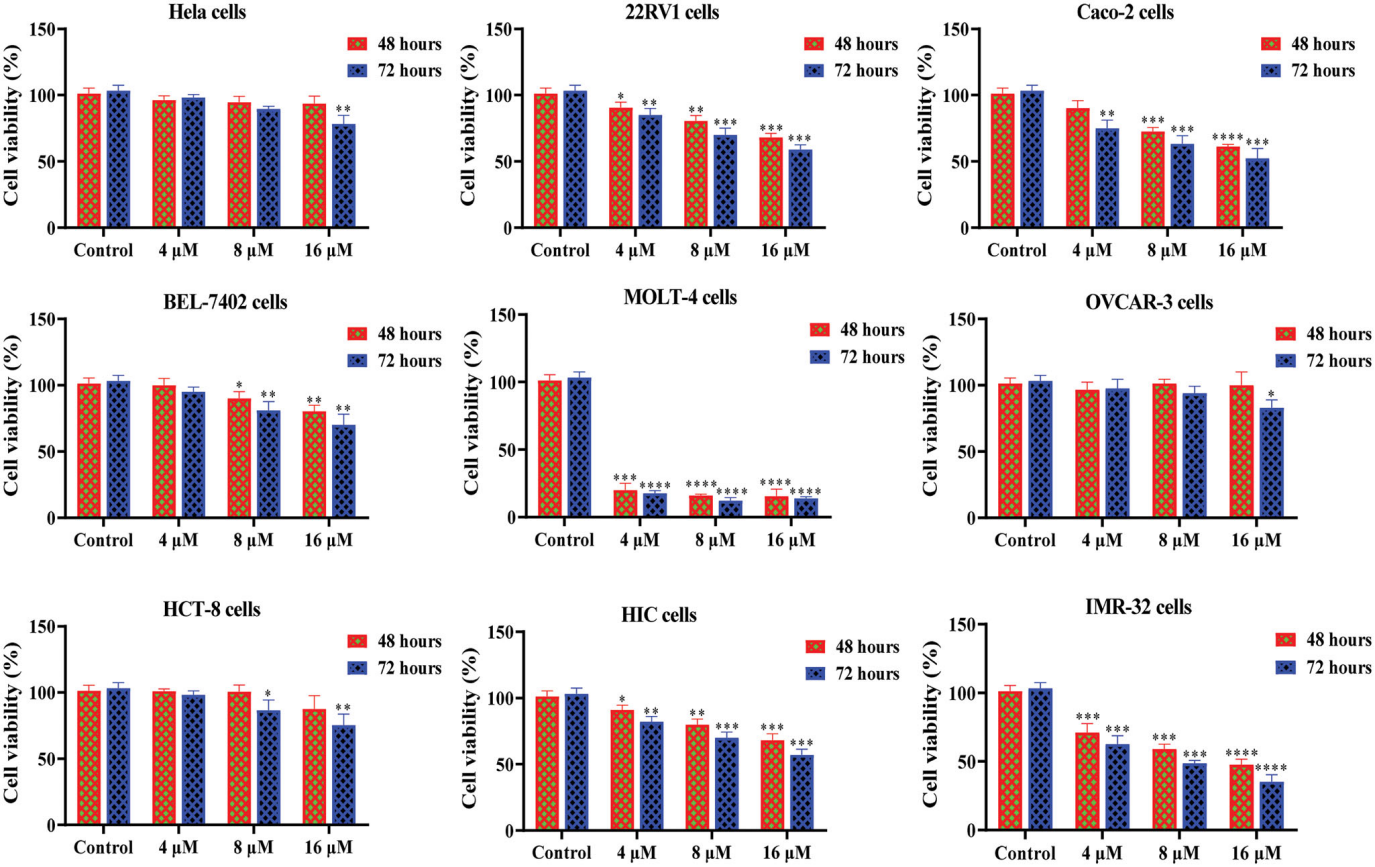
Cell viability of **D6** against different cancer cell lines for 48 h and 72 h. *P < 0.05, **P < 0.01, ***P < 0.001, and ****P < 0.0001 were considered statistically significant compared with the control.

Based on the screening activity results of all synthetic derivatives, the most potent chalcone **D6** was prioritised to perform further experiments for evaluating its antiproliferative potential. In addition, Hela (cervical cancer cells), 22RV1 (prostate cancer cells), Caco-2 (colon cancer cells), BEL-7402 (liver cancer cells), MOLT-4 (leukaemia cells), OVCAR-3 (ovarian cancer cells), HCT-8 (cecal adenocarcinoma cells) and IMR-32 (neuroblastoma cells) were treated with compound **D6** at different concentrations (control, 4 μM, 8 μM, and 16 μM). As shown in Figure 4, chalcone **D6** displayed the potential antiproliferative effects against 22RV1, Caco-2, MOLT-4 and IMR-32 cells. Among them, chalcone **D6** showed the most potent antiproliferation efficiency around 80% for 48 h at 4 μM against leukaemia MOLT-4 cells. These results indicated that chalcone **D6** might be a broad-spectrum antitumor agent.

### Chalcone D6 exhibited the potently antitumor activity *in vitro* against leukaemia cells

**Figure 5.**
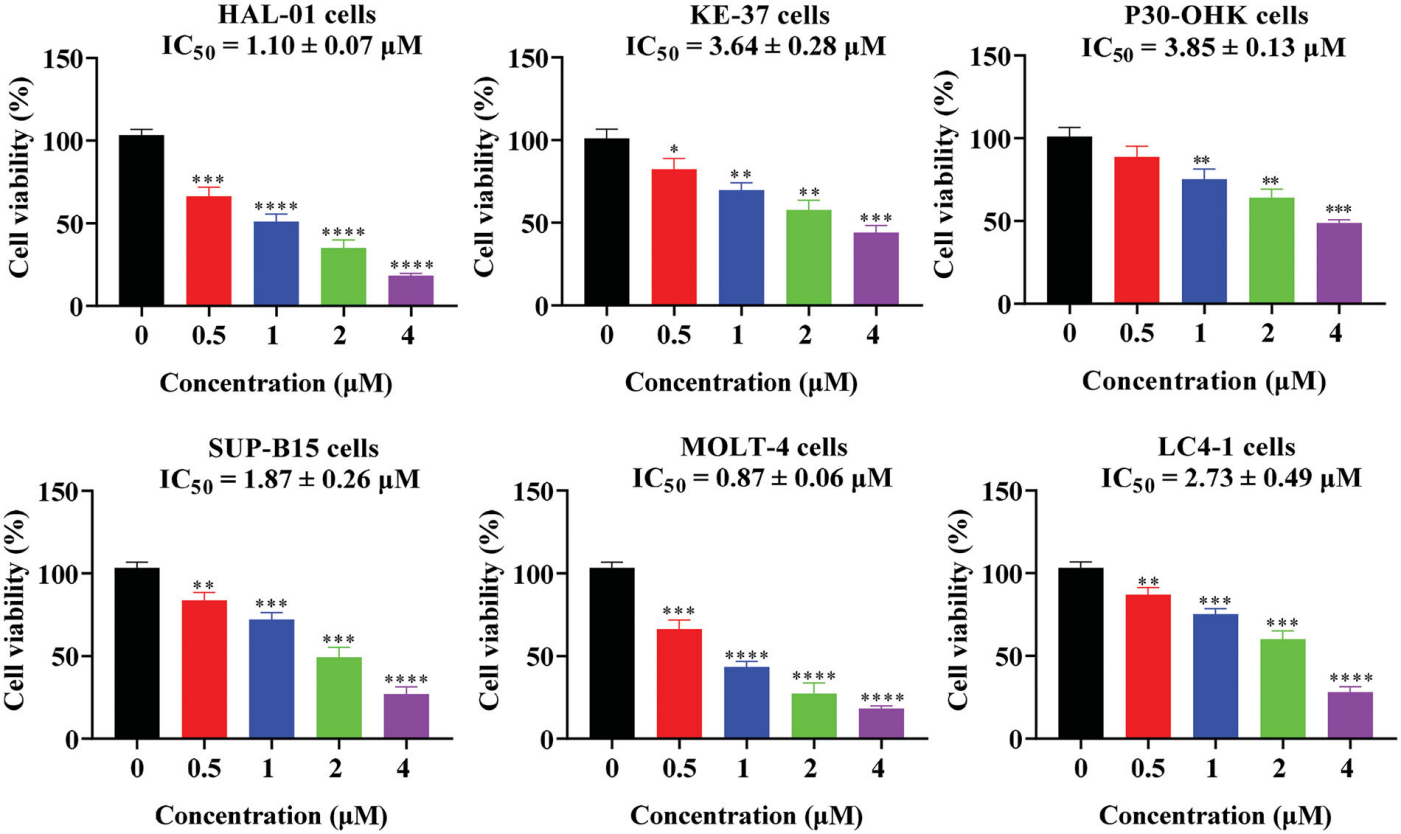
Antitumor activity *in vitro* of **D6** against leukaemia cells. *P < 0.05, **P < 0.01, ***P < 0.001, and ****P < 0.0001 were considered statistically significant compared with the control.

Based on the antiproliferative results, concentrations of 0.5 μM, 1 μM, 2 μM, and 4 μM were chosen to investigate the proliferation effects of chalcone **D6** on the cell viability of leukaemia cells (HAL-01, KE-37, P30-OHK, SUP-B15, MOLT-4 and LC4-1). We added the 5-Fluorouracil as a positive control to do the cytotoxicity assays in these leukaemia cells. The IC_50_ values of 5-Fluorouracil against HAL-01, KE-37, P30-OHK, SUP-B15, MOLT-4 and LC4-1 cells were 5.10 μM, 4.02 μM, 4.17 μM, 2.10 μM, 2.89 μM and 10.2 μM, respectively. From the results of Figure 5, chalcone **D6** inhibited cell proliferation with IC_50_ values of 1.10 μM, 3.64 μM, 3.85 μM, 1.87 μM, 0.87 μM and 2.73 μM against HAL-01, KE-37, P30-OHK, SUP-B15, MOLT-4 and LC4-1 leukaemia cells. These findings supported that chalcone **D6** potently inhibited cell proliferation against leukaemia cells in a concentration dependent manner.

### Chalcone D6 selectively inhibited LSD1 in a time dependent and reversible manner

**Figure 6.**
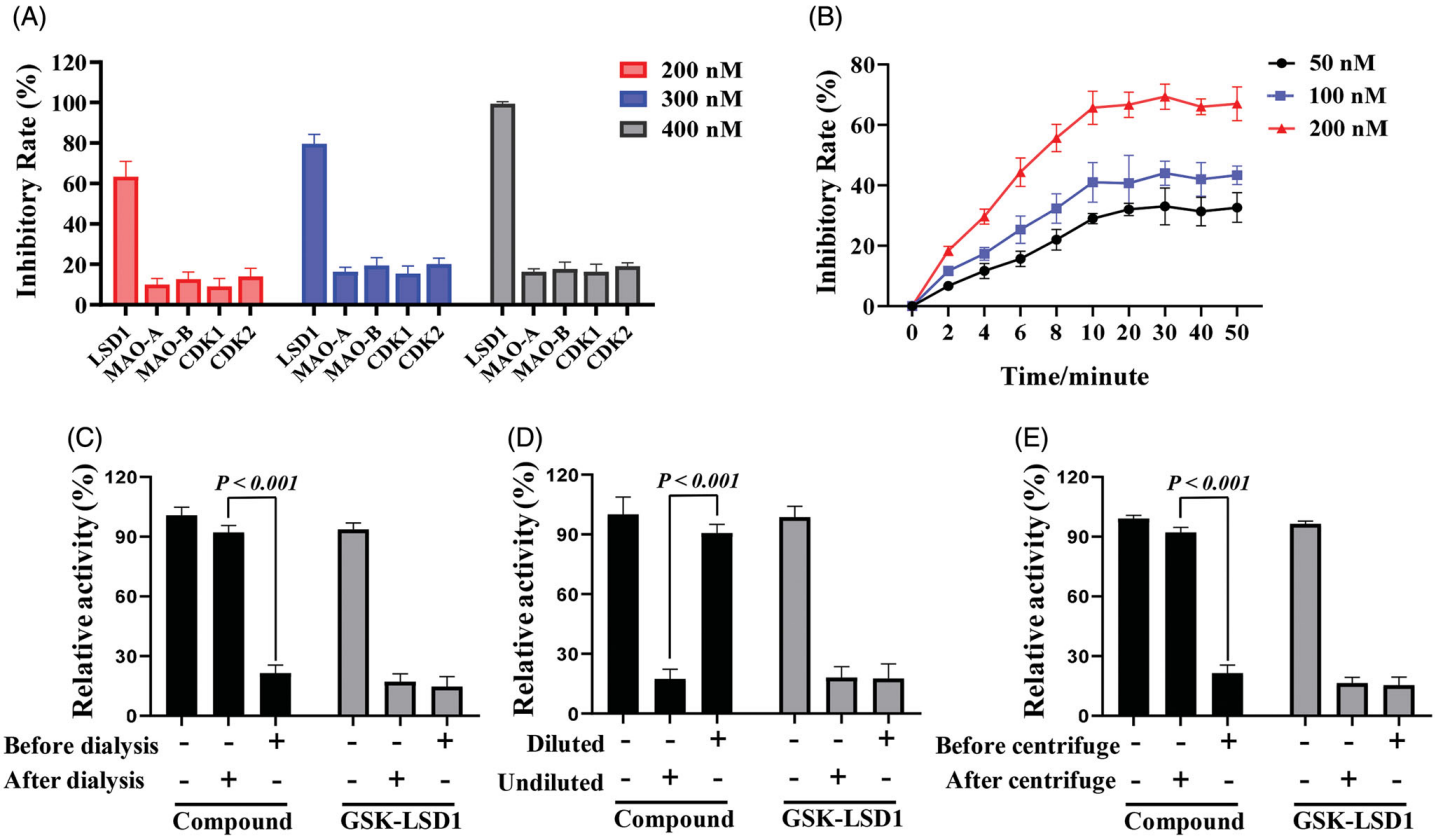
(A) *In vitro* enzyme inhibition of **D6** (200 nM, 300 nM and 400 nM) against LSD1, MAO-A/B and CDK1/2. (B) Inhibitory Rates of **D6** against LSD1. (C, D, E) The dialysis experiments, dilution assay and ultrafiltration experiments of **D6**. GSK-LSD1 was used as a positive control.

LSD1 belonged to the FAD dependent monoamine oxidases family including MAO-A and MAO-B, and shared the similar enzymatic mechanism of MAO-A/B^30^. In addition, chalcone-based analogues represented a privileged scaffold for developing inhibitors of cyclin-dependent kinases^31^. Thus, the selectivity of chalcone **D6** against homologous proteins MAO-A/B and CDK1/2 was explored in this work. As shown in Figure 6(A), chalcone **D6** at 400 nM weakly inhibited MAO-A, MAO-B, CDK1 and CDK2 with the rates of only 16.3%, 17.7%, 16.3% and 19.0%, respectively, while it showed about 99.3% of inhibition against LSD1. These findings indicated the high selectivity of chalcone **D6** on LSD1 *in vitro*. Then, time-dependent experiments were also performed and results showed that chalcone **D6** inhibited LSD1 in a time dependent manner (Figure 6(B)). Importantly, the dialysis experiment (Figure 6(C)) and dilution assay (Figure 6(D)) indicating that chalcone **D6** was a reversible LSD1 inhibitor. To further confirm the potential binding manner of chalcone **D6** against LSD1 recombinant, the centrifuge experiment was also carried out. With the aid of 10 kDa ultracentrifuge filter, reversible compound was supposed to be removed from LSD1 by centrifuge. So, chalcone **D6** was characterised as a reversible LSD1 inhibitor as split of chalcone **D6** by ultracentrifuge may rescue the activity of LSD1 (Figure 6(E)). All these results showed that chalcone **D6** could selectively inhibit LSD1 in a time dependent and reversible manner.

### Antiproliferative effects of chalcone D6 against MOLT-4 LSD1 knockdown cells

**Figure 7.**
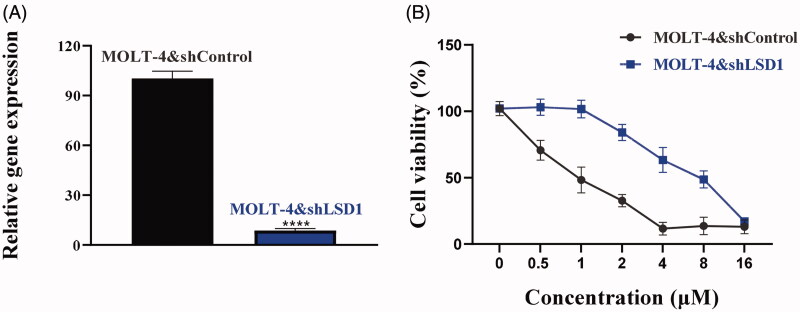
(A) The gene expression of LSD1 in MOLT-4&shLSD1 cells and MOLT-4&shControl cells. (B) Antiproliferative effects of **D6** against MOLT-4&shLSD1 cells and MOLT-4&shControl cells. ****P < 0.0001 was considered statistically significant compared with the control.

LSD1 was aberrantly over-expressed in leukaemia cells, and associated with tumorigenesis^32^. In view of the inhibitory potency against LSD1, chalcone **D6** was chosen for further antiproliferative studies. In this work, we used the LSD1 knock-down MOLT-4 cells (MOLT-4&shLSD1) and control cells (MOLT-4&shControl) to investigate its antiproliferative activity. Firstly, the gene expression of LSD1 in MOLT-4&shLSD1 cells and MOLT-4&shControl cells was detected by quantitative real-time PCR, the results were shown in Figure 7(A). With these two cell lines in hand, we nextly used the MTT assay to examine the antiproliferative effects of chalcone **D6** against MOLT-4&shLSD1 cells and MOLT-4&shControl cells. As shown in Figure 7(B), chalcone **D6** significantly suppressed the proliferation of MOLT-4&shControl cells in a concentration dependent manner with the IC_50_ value of 0.89 μM. In contrast, **D6** inhibited MOLT-4&shLSD1 cells with the IC_50_ value of 7.83 μM, about 8 ∼ 9-fold less potent against MOLT-4&shControl cells. The activity discrepancy observed indicated that the antiproliferative effects of chalcone **D6** against MOLT-4 cells were dependent on LSD1 inhibition, and also suggested that chalcone **D6** was cellularly active against LSD1, excluding off-target effects.

### Chalcone D6 regulated the expression of LSD1 substrates H3K9me1/2

**Figure 8.**
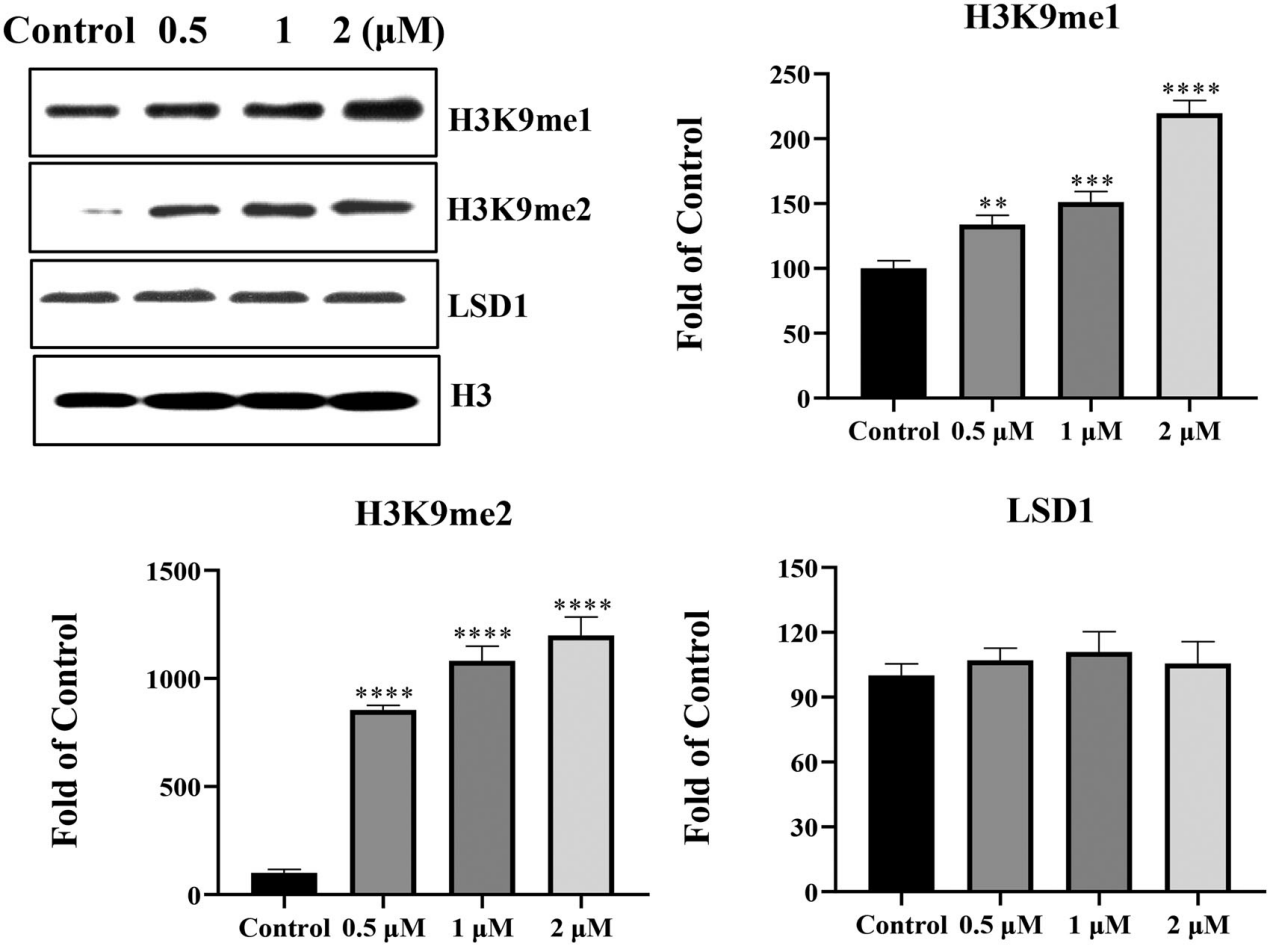
The amount of H3K9me1 and H3K9me2 in MOLT-4 cells treated by chalcone **D6**. **P < 0.01, ***P < 0.001 and ****P < 0.0001 were considered statistically significant compared with the control.

To further determine the inhibitory effects of chalcone **D6** against LSD1 in MOLT-4 cells, amount of two reported LSD1 substrates H3K9me1 and H3K9me2 were analysed by western blotting experiments. As shown in Figure 8, the amount of H3K9me1 and H3K9me2 showed a concentration dependent accumulation in the presence of chalcone **D6**. In addition, the treatment of **D6** in MOLT-4 cells did not affect the expression level of LSD1. Collectively, these results suggested that chalcone **D6** is a cellular active LSD1 inhibitor in leukaemia MOLT-4 cells.

### Molecular docking of chalcone D6

**Figure 9.**
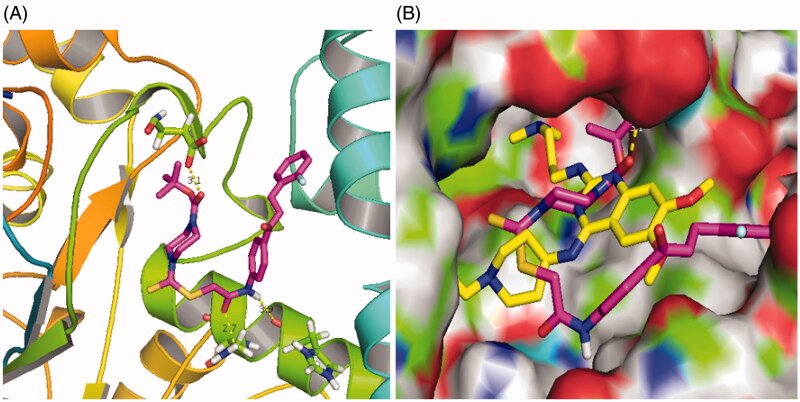
Molecular docking studies of chalcone **D6**. (A) The complex formed between LSD1 and compound **D6**. (B) A similar pocket between the reference compound **E11** (yellow structure) and **D6** (magenta structure).

Based on the above experiments, chalcone **D6** has been identified as a novel LSD1 inhibitor. In the current work, molecular docking methodologies were also used to explore any molecular interaction exist between chalcone **D6** and residues lies in the active site cativity of LSD1. We have used Autodock as an automated tool to perform docking and selected PDB code 5l3e (Resolution: 2.80 Å). As shown in Figure 9, chalcone **D6** formed three hydrogen bonds with residues His532, Asn535 and Asp556, respectively. In addition, chalcone **D6** formed hydrophobic effects with residues Leu386, Phe382 and Phe538. These results explained that chalcone scaffold was a promising unit for targeting LSD1. Based on the reported reference [33], **E11** as a reference compound was docked using the same protocol and compared with chalcone **D6**. In the Figure 9, the reference compound **E11** (yellow structure) was docked into a similar pocket as chalcone **D6** (magenta structure).

### Xenograft study of chalcone D6

**Figure 10.**
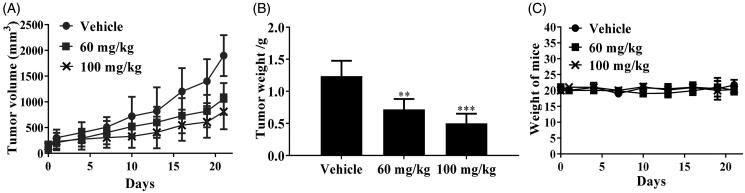
*In vivo* anticancer effects of chalcone **D6**. (A) Tumour volume. (B) Tumour weight. **C** Weight of mice. **P < 0.01 and ***P < 0.001 were considered statistically significant compared with the control.

Since the potently antiproliferative activity of chalcone **D6** against MOLT-4 cells, we also evaluated the anticancer effects of chalcone **D6** on xenograft models bearing MOLT-4 cells. After the treatment of chalcone **D6** (60 mg/kg and 100 mg/kg), the body weight of mice, the tumour weight and the tumour volume were measured and recorded. As shown in Figure 10, chalcone **D6** inhibited tumour growth remarkably, while the body weight was almost unchanged, suggesting the antitumor efficacy and low global toxicity.

## Conclusion

A series of chalcone derivatives were designed, synthesised and evaluated for LSD1 inhibitory activity. All chalcone-dithiocarbamate hybrids **D1**∼**D7** exhibited potentially inhibitory activity against LSD1. Especially, chalcone **D6** showed the best LSD1 inhibitory activity with an IC_50_ value of 0.14 μM. In addition, **D6** inhibited cell proliferation with IC_50_ values of 1.10 μM, 3.64 μM, 3.85 μM, 1.87 μM, 0.87 μM and 2.73 μM against HAL-01, KE-37, P30-OHK, SUP-B15, MOLT-4 and LC4-1 leukaemia cells. Further investigations demonstrated that compound **D6** selectively inhibited LSD1 in a time dependent and reversible manner. It also up-regulated the expression levels of H3K9me1 and H3K9me2 against MOLT-4 cells. Importantly, chalcone **D6** inhibited *in vivo* tumour growth in a xenograft model without apparent toxicity. Taken together, chalcone **D6** could be a lead candidate for its further development in the treatment of leukaemia.

## Supplementary Material

Supplemental Material
